# Análise Têmporo-Espacial da Mortalidade por Cardiomiopatias no Brasil entre 2001 e 2021

**DOI:** 10.36660/abc.20260064

**Published:** 2026-07-15

**Authors:** Luiz Fernando Kubrusly, Ana Clara Abdo Lopes, Fernanda Ritt de Souza, Gustavo Ferrucio Dieter, Laura Nadolny, Leonardo Moreira Dias, Letícia Moreira Dias, Luis Gustavo Machado Farias, Roberto Oliveira Basso, Vitória Naomi Okimura

**Affiliations:** 1 Faculdade Evangélica Mackenzie do Paraná Curitiba PR Brasil Faculdade Evangélica Mackenzie do Paraná, Curitiba, PR – Brasil; 2 Faculdade Pequeno Príncipe Curitiba PR Brasil Faculdade Pequeno Príncipe, Curitiba, PR – Brasil

**Keywords:** Cardiomiopatias, Mortalidade, Análise Espaço-Temporal, Epidemiologia

## Abstract

**Fundamento:**

O Brasil apresenta variações regionais na mortalidade por cardiomiopatias, influenciada por características demográficas, desigualdades socioeconômicas e diferenças no acesso aos serviços de saúde. Entre 2001 e 2021, foram registrados 272.448 óbitos pela doença no país.

**Objetivo:**

Analisar, em nível nacional, o perfil epidemiológico e a distribuição têmporo-espacial da mortalidade por cardiomiopatias no Brasil, no período de 2001 a 2021.

**Métodos:**

Estudo epidemiológico baseado em dados secundários do Sistema de Informação sobre Mortalidade (SIM/DATASUS) e estimativas populacionais do IBGE. Calcularam-se taxas brutas de mortalidade por município. A análise temporal foi realizada pelo Joinpoint, estimando-se a variação percentual anual (APC). A análise espacial utilizou o GeoDa, com suavização bayesiana e avaliação da autocorrelação espacial pelos índices de Moran Global e LISA. A significância estatística foi estabelecida em p < 0,05.

**Resultados:**

Ocorreram 272.448 óbitos por cardiomiopatias, predominando em homens (58,65%), indivíduos com 80 anos ou mais (25,74%), brancos (52,18%), casados (36,77%) e com 1 a 3 anos de estudo completos (21,91%). A mortalidade média foi de 6,66 por 100 mil habitantes, atingindo 7,64 por 100 mil em 2004 e caindo para 4,15 por 100 mil em 2020. Observou-se redução anual significativa de 1,86% (p<0,05). A análise espacial revelou aglomerados de alta mortalidade principalmente no Centro-Oeste, Sul e Sudeste, com destaque para Corumbá de Goiás (49,34/100 mil).

**Conclusão:**

Houve redução significativa da mortalidade por cardiomiopatias, embora persistam áreas críticas que exigem estratégias direcionadas para vigilância, diagnóstico precoce e manejo adequado.

## Graphical abstract

**Figura Central:**
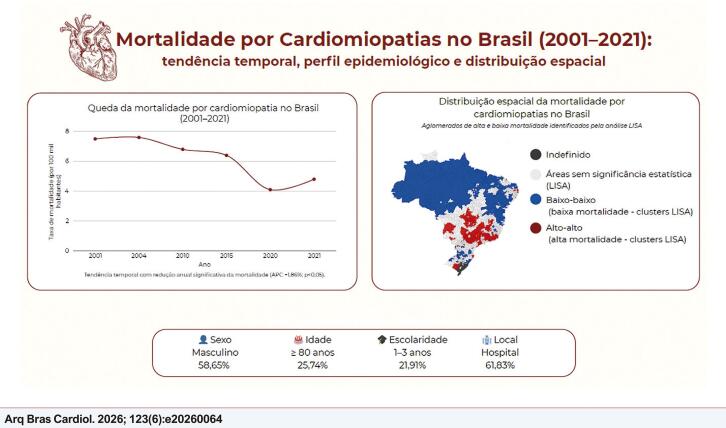
Análise Têmporo-Espacial da Mortalidade por Cardiomiopatias no Brasil entre 2001 e 2021

## Introdução

As cardiomiopatias constituem um grupo heterogêneo de doenças do miocárdio, caracterizadas por alterações estruturais e funcionais que podem evoluir para insuficiência cardíaca, arritmias malignas e morte súbita. Segundo a Classificação Internacional de Doenças (CID-10), essas patologias estão classificadas sob o código I42. Nas últimas décadas, observou-se aumento da prevalência global dessas doenças, especialmente da cardiomiopatia dilatada, cuja incidência varia entre 5 e 10 casos por 100.000 habitantes ao ano, refletindo o envelhecimento populacional e a maior carga de doenças crônicas não transmissíveis.^1-3^

A etiologia das cardiomiopatias é multifatorial, envolvendo causas inflamatórias, tóxicas, metabólicas e genéticas, com destaque para mutações hereditárias, particularmente na cardiomiopatia hipertrófica.^3,4^ Fatores de risco como hipertensão arterial, diabetes mellitus, obesidade e consumo excessivo de álcool também estão associados ao desenvolvimento e à progressão dessas condições.^5^

Clinicamente, os pacientes podem apresentar dispneia, fadiga, palpitações e edema periférico, com manifestações que variam desde formas assintomáticas até quadros graves de insuficiência cardíaca ou morte súbita.^6,7^ O diagnóstico baseia-se na avaliação clínica associada a exames complementares, sendo o ecocardiograma considerado o padrão-ouro para a avaliação da função ventricular e da morfologia cardíaca.^8^ O tratamento é direcionado à etiologia subjacente e inclui terapias farmacológicas e intervenções não farmacológicas. Apesar dos avanços terapêuticos, complicações e mortalidade permanecem relevantes.^9,10^

No Brasil, estudos evidenciam variações regionais expressivas na mortalidade por cardiomiopatias, sugerindo influência de fatores demográficos, socioeconômicos e desigualdades no acesso aos serviços de saúde.^11-13^ Entretanto, grande parte das investigações apresenta recortes temporais limitados ou análises regionais isoladas, dificultando a compreensão integrada da distribuição espacial e da tendência temporal da mortalidade em âmbito nacional.^14,15^

Embora estudos prévios frequentemente relatem diferenças regionais utilizando dados agregados, a análise espaço-temporal oferece uma abordagem mais abrangente ao avaliar simultaneamente variações no espaço e no tempo. Isso permite a identificação de clusters persistentes ou emergentes e uma melhor compreensão dos padrões geográficos dinâmicos.^16,17^

Além disso, questões metodológicas como a agregação espacial (problema da unidade de área modificável — MAUP) e o viés ecológico podem influenciar os resultados, reforçando a importância de estratégias analíticas adequadas em epidemiologia espacial.^18^ Técnicas de suavização bayesiana e outros métodos de estabilização de taxas também contribuem para melhorar a confiabilidade das estimativas em pequenas áreas, ao reduzir flutuações aleatórias.^19^

Nesse contexto, o presente estudo teve como objetivo analisar o perfil epidemiológico e a distribuição espaço-temporal da mortalidade por cardiomiopatias no Brasil entre 2001 e 2021. Como objetivos secundários, buscou-se identificar padrões espaciais e clusters de mortalidade, bem como descrever a distribuição dos óbitos segundo características sociodemográficas. Em acréscimo, o local de ocorrência dos óbitos foi analisado como uma variável descritiva para complementar o perfil epidemiológico.

## Métodos

Trata-se de um estudo epidemiológico de análise de dados secundários em saúde com enfoque na análise têmporo-espacial. Os dados referentes aos casos de óbito por cardiomiopatias foram obtidos no Sistema de Informação sobre Mortalidade (SIM), do Departamento de Informática do Sistema Único de Saúde (DATASUS) do Ministério da Saúde (MS), via Tabnet, por meio do local de óbito e local de ocorrência. Os dados populacionais utilizados como denominadores para o cálculo das taxas de mortalidade foram obtidos do DATASUS, com base nas estimativas populacionais do IBGE por município, sexo e faixa etária para o período de 2000 a 2021. Essas estimativas são derivadas de métodos de projeção demográfica e ajustes intercensitários realizados pelo IBGE, utilizando o censo nacional de 2010 como referência para garantir a consistência da série temporal.

Para a análise da tendência de mortalidade no Brasil, foi selecionado o período de 2001 a 2021, visando melhor compreensão do desfecho nessa série histórica. Trata-se de uma análise censitária, sem seleção individual de participantes, que incluiu a totalidade dos óbitos por cardiomiopatias registrados no SIM entre 2001 e 2021, considerando-se como população os indivíduos de todas as idades. Consideraram-se para a análise as variáveis sexo, faixa etária, cor/raça, estado civil, escolaridade, óbitos por residência, ano do óbito, local do óbito e local de ocorrência. As categorias de escolaridade seguiram a classificação disponível na base de dados do DATASUS e foram mantidas conforme originalmente fornecidas, a fim de garantir consistência com os sistemas nacionais de informação em saúde. A variável raça/cor foi categorizada conforme a classificação padronizada adotada pelo DATASUS/IBGE.

Para a causa básica desses óbitos, foi adotado o CID I42 da Classificação Internacional de Doenças (CID-10). As informações do perfil epidemiológico e frequência de óbitos foram compiladas no software Microsoft Excel para Microsoft 365 MSO. As variáveis nominais foram analisadas por meio da frequência absoluta e percentual de ocorrência na população em estudo. A mortalidade anual foi calculada considerando-se o número total de óbitos no estado como numerador e a população do estado naquele ano como denominador, expressa por 100 mil habitantes. As taxas de mortalidade municipais foram estimadas por meio de uma abordagem baseada na média populacional, definida como o número médio de óbitos no período dividido pela população média entre 2001 e 2021, expressa por 100 mil habitantes. A população média foi obtida pela soma das estimativas populacionais anuais ao longo do período estudado, dividida pelo número de anos. Essa abordagem foi adotada para reduzir flutuações anuais e fornecer estimativas mais estáveis para análises em pequenas áreas; no entanto, não considera diferenças na estrutura etária entre os municípios, o que pode influenciar as comparações.

**Figura 1:**
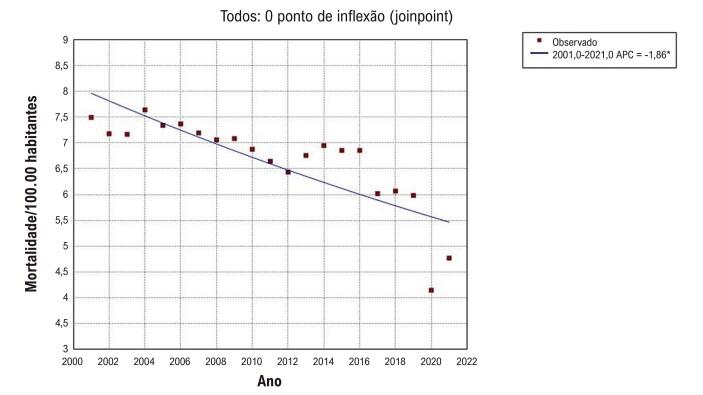
– Padrão temporal da mortalidade por cardiomiopatia no Brasil entre 2001 e 2021. * Indica que a Variação Percentual Anual (APC) é significativamente diferente de zero no nível alfa = 0,05. Modo Final Selecionado: 1 Ponto de inflexão (Joinpoint). Fonte: Os autores (2025).

**Figura 2:**
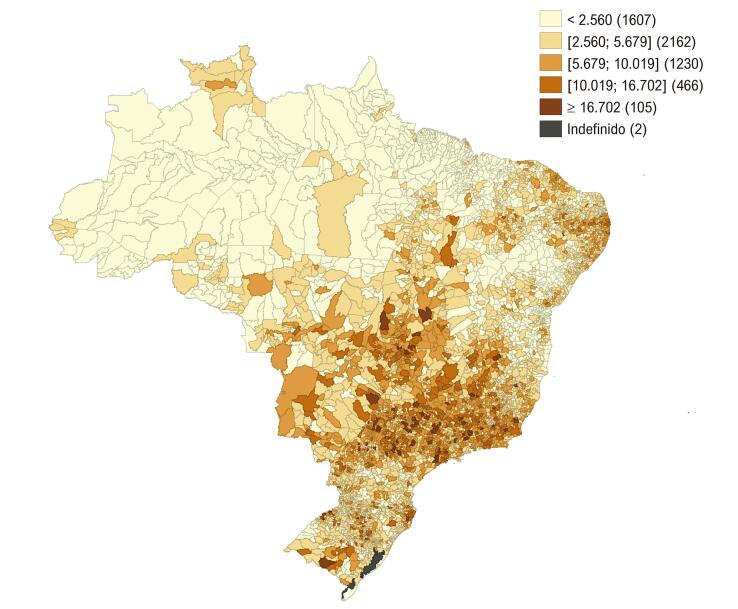
– Taxa de mortalidade bruta por cardiomiopatia no Brasil, entre 2001 e 2021. Fonte: Os autores (2025).

O desfecho primário do estudo foi a mortalidade por cardiomiopatias, definida a partir do código I42 da Classificação Internacional de Doenças (CID-10). Inicialmente, realizou-se a análise da tendência temporal da mortalidade por anos (Figura 1). Assim, foi avaliada a variação percentual anual (*annual percent change* – APC) da tendência estudada, com intervalo de confiança de 95% (IC95%) e significância estatística p<0,05. A análise de tendência temporal foi realizada utilizando o programa Joinpoint Regression Program (versão 5.0.2, National Cancer Institute, EUA), com regressão por mínimos quadrados ponderados, estimando a variação percentual anual (APC) e intervalos de confiança de 95%. Foi ajustado um modelo log-linear, e o número de pontos de inflexão (*joinpoints*) foi determinado por meio de testes de permutação, de acordo com as configurações padrão do software. O modelo final selecionado não incluiu pontos de inflexão (ou seja, apresentou uma única tendência temporal). Em seguida, foi realizada a análise espacial da distribuição da mortalidade por cardiomiopatias para cada região do Brasil (Figura 2).

A suavização espacial foi realizada utilizando o método bayesiano empírico local, com o objetivo de reduzir flutuações aleatórias nas taxas de mortalidade municipais. Foi construída uma matriz de pesos espaciais baseada na contiguidade do tipo “*queen*” de primeira ordem, na qual municípios que compartilham pelo menos uma fronteira ou vértice comum foram considerados vizinhos.

Os limites municipais foram definidos de acordo com a divisão territorial oficial fornecida pelo Instituto Brasileiro de Geografia e Estatística (IBGE), sem qualquer modificação. Os dados de mortalidade do DATASUS foram vinculados aos municípios por meio dos códigos oficiais do IBGE.

Análises de sensibilidade utilizando definições alternativas de vizinhança (como contiguidade do tipo “*rook*”) não foram realizadas. No entanto, a escolha da contiguidade *queen* foi adotada por apresentar uma estrutura de vizinhança mais abrangente, amplamente utilizada em estudos epidemiológicos espaciais e que fornece estimativas mais estáveis em áreas com configurações territoriais heterogêneas.

**Figura 3:**
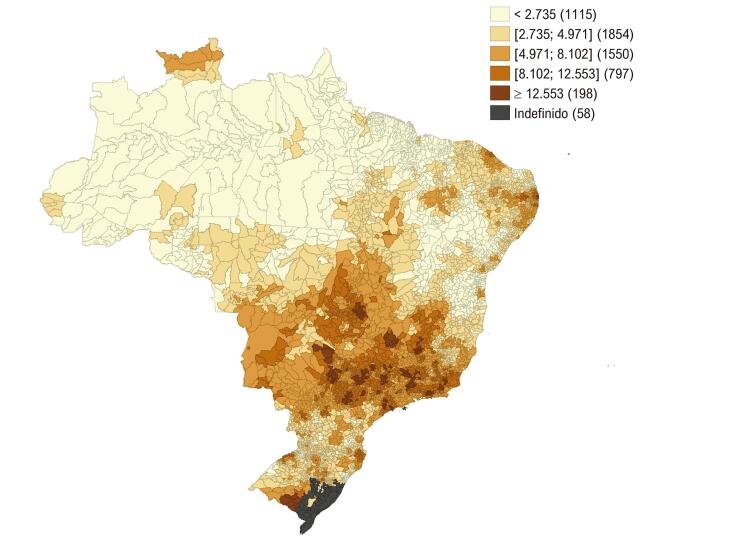
– Taxa de mortalidade suavizada pelo método bayesiano empírico local por cardiomiopatia no Brasil entre 2001 e 2021. Fonte: Os autores (2025).

**Figura 4:**
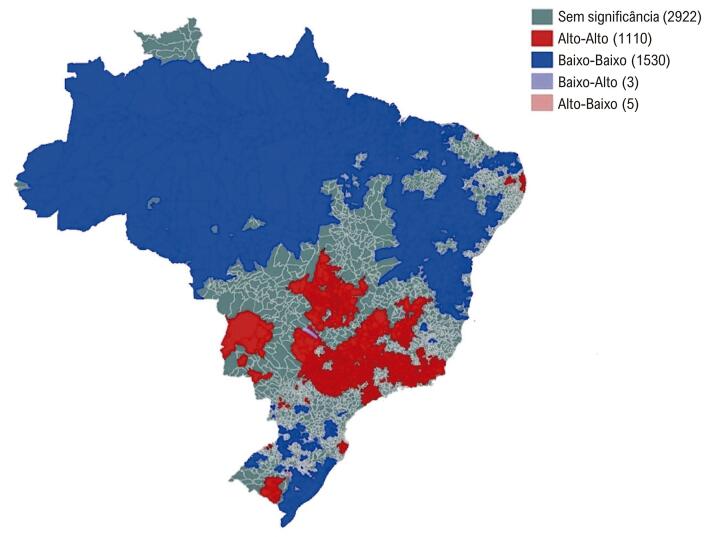
– Aglomerados espaciais de mortalidade por cardiomiopatia no Brasil entre 2001 e 2021. Fonte: Os autores (2025).

**Figura 5:**
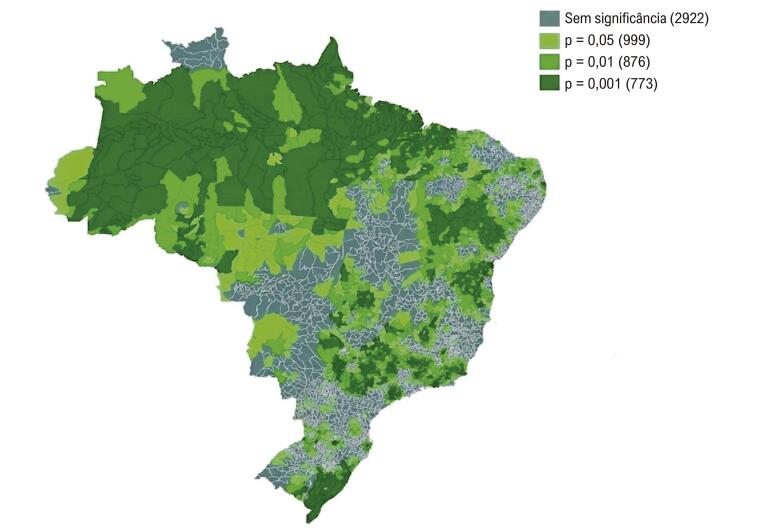
– Significância estatística dos aglomerados espaciais. Fonte: Os autores (2025).

As análises espaciais e a construção dos mapas temáticos foram realizadas utilizando o software GeoDa, versão 1.22.0.4 (2023). A categorização das legendas dos mapas seguiu diferentes abordagens conforme o tipo de análise realizada. Para as taxas brutas de mortalidade (Figuras 1 e 2), os valores foram classificados por quantis, permitindo uma distribuição equilibrada dos municípios entre as categorias e melhorando a visualização e a comparabilidade. Para as taxas suavizadas obtidas pelo método bayesiano empírico local (Figuras 3–5), a classificação refletiu a distribuição dos valores ajustados, com o objetivo de evidenciar padrões espaciais após a redução das flutuações aleatórias. Essas estratégias de classificação são comumente utilizadas em análises espaciais exploratórias; no entanto, diferentes métodos de categorização podem levar a variações na interpretação visual dos padrões espaciais.

Para a identificação de aglomerados espaciais, utilizou-se o Índice de Moran Global e Local. O Índice de Moran Global mede a correlação entre vizinhos de primeira ordem e foi usado para testar a hipótese de dependência espacial. O método identifica a autocorrelação espacial e pode variar entre -1 e +1, no qual os valores próximos a zero indicam ausência de dependência espacial, considerando-se significante p<0,05. Caso a hipótese de dependência seja aceita, utiliza-se o Índice de Moran Local (LISA, do inglês *Local Index of Spatial Association*) para observar a presença de agregados espaciais, dado p<0,05. Os resultados das análises descritas acima foram demonstrados pelo Moran Map e LISA Map (Figuras 4 e 5). O Moran Map demonstra graficamente o grau de similaridade entre vizinhos, sendo representado por quatro quadrantes:

1. Alto-alto (quadrante superior direito): Correspondem a municípios que possuem altas taxas de mortalidade e estão próximos a municípios que também possuem altas taxas de mortalidade.
2. Baixo-baixo (quadrante inferior esquerdo): Correspondem a municípios que possuem baixas taxas de mortalidade e estão próximos a municípios que também possuem baixas taxas de mortalidade.
3. Alto-baixo (quadrante inferior direito): Correspondem a municípios que possuem altas taxas de mortalidade e estão próximos a municípios que possuem baixas taxas de mortalidade.
4. Baixo-alto (quadrante superior esquerdo): Correspondem a municípios que possuem baixas taxas de mortalidade e estão próximos a municípios que possuem altas taxas de mortalidade.

Os dados utilizados são de acesso público, agregados e sem identificação individual, não havendo a possibilidade de danos físicos ou morais na perspectiva do indivíduo e da coletividade. Desse modo, o estudo dispensa apreciação por Comitê de Ética em Pesquisa, estando em conformidade com a Resolução nº 466/2012 do Conselho Nacional de Saúde. Além disso, haja vista que o estudo é baseado em dados secundários, os achados podem refletir limitações relacionadas à qualidade da informação, como sub-registro e dados incompletos nas declarações de óbito.

## Resultados

### Perfil epidemiológico dos dados

Entre 2001 e 2021, foram registrados no Brasil 272.448 óbitos por cardiomiopatias. Como é mostrado na Figura Central, a caracterização sociodemográfica evidenciou maior ocorrência de óbitos entre indivíduos do sexo masculino e com idade igual ou superior a 80 anos. Observou-se ainda predomínio entre pessoas autodeclaradas brancas, casadas e com nível de escolaridade entre 1 a 3 anos. O perfil epidemiológico detalhado dos óbitos encontra-se descrito na Tabela 1.

**Tabela 1:** – Perfil epidemiológico dos indivíduos que foram a óbito por cardiomiopatia no Brasil entre 2001 e 2021

Variável	n	%
**Sexo**		
Masculino	159.782	58,65
Feminino	112.618	41,33
Ausente	48	0,02
**Faixa etária (anos)**		
<1	2.611	0,96
1-4	1.327	0,49
5-9	572	0,21
10-14	834	0,31
15-19	1.664	0,61
20-29	5.635	2,07
30-39	12.571	4,61
40-49	24.747	9,08
50-59	39.897	14,64
60-69	51.505	18,90
70-79	59.978	22,01
≥80	70.132	25,75
Idade ausente	975	0,36
**Cor/raça**		
Branca	142.166	52,18
Preta	25.990	9,54
Parda	86.935	31,91
Amarela	1.341	0,49
Indígena	380	0,14
Ausente	15.636	5,74
**Estado civil**		
Solteiro	66.998	24,59
Casado	100.189	36,77
Viúvo	66.280	24,33
Separado judicialmente	15.681	5,76
Outro	3.344	1,23
Ausente	19.956	7,32
**Escolaridade (anos)**		
Nenhuma	39.515	14,50
1-3	59.684	21,91
4-7	56.125	20,60
8-11	29.699	10,90
≥12	10.933	4,01
Ausente	76.492	28,08
**Local de ocorrência do óbito**		
Hospital	168.444	61,83
Outro estabelecimento de saúde	16.890	6,20
Domicílio	72.962	26,78
Via pública	4.852	1,78
Outros	8.948	3,28
Ausente	352	0,13

Fonte: Departamento de Informática do Sistema Único de Saúde (DATASUS), Ministério da Saúde, Brasil.

Em relação ao local de ocorrência, a maioria dos óbitos ocorreu em ambiente hospitalar, seguida por óbitos em domicílio, enquanto as demais categorias apresentaram menor frequência relativa (Tabela 1).

### Análise temporal da mortalidade

A taxa média de mortalidade por cardiomiopatias durante o período do estudo foi de 6,66 óbitos por 100.000 habitantes, sendo a maior taxa observada em 2004 e a menor em 2020. A análise de tendência temporal demonstrou uma redução significativa da mortalidade ao longo do período estudado, com uma diminuição média anual de 1,86% (APC = −1,86%; IC 95% [−2,73; −1,03], p < 0,001). O modelo final não incluiu pontos de inflexão (joinpoints), indicando uma tendência constante de queda ao longo do tempo, conforme demonstrado na Figura Central e na Figura 1.

### Análise temporal da mortalidade

A taxa média de mortalidade por cardiomiopatias durante o período do estudo foi de 6,66 óbitos por 100.000 habitantes, sendo a maior taxa observada em 2004 e a menor em 2020. A análise de tendência temporal demonstrou uma redução significativa da mortalidade ao longo do período estudado, com uma diminuição média anual de 1,86% (APC = −1,86%; IC 95% [−2,73; −1,03], p < 0,001). O modelo final não incluiu pontos de inflexão (joinpoints), indicando uma tendência constante de queda ao longo do tempo, conforme demonstrado na Figura Central e na Figura 1.

### Análise espacial da mortalidade

A análise espacial da taxa bruta de mortalidade evidenciou heterogeneidade na distribuição dos óbitos por cardiomiopatias no território nacional, com maiores taxas concentradas predominantemente nas regiões Centro-Oeste, Sul e Sudeste (Figura 2). Os municípios com as maiores taxas de mortalidade no período incluíram Corumbá de Goiás, São José do Rio Pardo, Itajobi, Santo Inácio e São Manuel.

Após a aplicação da suavização bayesiana empírica local, observou-se um padrão espacial mais definido, com agregação de municípios com elevadas taxas de mortalidade principalmente nas regiões Centro-Oeste e Sudeste do Brasil (Figura 3). A análise de autocorrelação espacial confirmou a presença de dependência espacial significativa, evidenciada pelo Índice de Moran Global positivo (I = 0,864; p = 0,01).

A análise local de autocorrelação (LISA) identificou aglomerados espaciais estatisticamente significativos. Os aglomerados do tipo alto-alto foram observados principalmente nas regiões Centro-Oeste, Sul e Sudeste, enquanto os aglomerados baixo-baixo concentraram-se predominantemente nas regiões Norte e Nordeste do país (Figura Central, Figuras 4 e 5).

## Discussão

Os achados deste estudo demonstram que, entre 2001 e 2021, foram registrados 272.448 óbitos por cardiomiopatias no Brasil, com predominância entre homens (58,65%), indivíduos com 80 anos ou mais (25,74%) e pessoas autodeclaradas brancas (52,18%). Esse perfil epidemiológico é consistente com dados previamente descritos na literatura, que apontam maior gravidade e letalidade das cardiomiopatias em populações idosas e do sexo masculino, associadas ao envelhecimento cardiovascular e à maior carga de comorbidades, como hipertensão arterial e diabetes mellitus.^2,5,20^

A maior mortalidade observada entre homens pode ser explicada, em parte, por diferenças biológicas e hormonais, além de maior exposição a fatores de risco e maior propensão a alterações estruturais do miocárdio e eventos cardiovasculares, como a morte súbita, o que está alinhado com achados de estudos internacionais e diretrizes clínicas.^2,6,7^ Ademais, referente à faixa etária, o envelhecimento populacional é um dos principais fatores de risco para o desenvolvimento da patologia, especialmente no caso da cardiomiopatia dilatada, devido ao aumento da vulnerabilidade miocárdica e da prevalência das comorbidades supracitadas, reforçando a relevância desse agravo em países com transição demográfica avançada, como o Brasil.^2,5,7,20^

Menor nível de escolaridade foi associado a maior mortalidade por cardiomiopatias: aproximadamente 21,91% dos indivíduos que faleceram devido a patologia tinham de 1 a 3 anos de estudo. Esse dado dialoga com o que foi identificado por Santos et al.,^14^ que sugere a influência dos determinantes sociais da saúde nos desfechos cardiovasculares, incluindo menor acesso a serviços especializados, diagnóstico tardio e menor adesão terapêutica. Além disso, a predominância de óbitos entre indivíduos casados (36,77%) pode refletir a maior longevidade observada nesse grupo, fenômeno já descrito em análises demográficas brasileiras.^21,22^

Quanto aos locais de ocorrência dos óbitos, a maior parte ocorreu em ambiente hospitalar (61,83%), o que pode indicar não só o aumento do acesso a internações, mas também o diagnóstico tardio das cardiomiopatias, haja vista que muitas vezes passam despercebidas nos estágios iniciais.^6^ Em contrapartida, a proporção significativa de mortes que ocorreram em domicílio (26,78%) sugere fragilidades no acompanhamento ambulatorial e no manejo precoce da patologia, especialmente em regiões com limitações geográficas e sociais importantes, conforme já destacado por Ziaeian e Fonarow.^2^

A redução observada na mortalidade por cardiomiopatias (diminuição média anual de 1,86%; p < 0,05) pode estar parcialmente relacionada aos avanços terapêuticos das últimas décadas, incluindo o uso de inibidores da enzima conversora de angiotensina, betabloqueadores, dispositivos implantáveis e transplante cardíaco. No entanto, o acesso a essas terapias permanece heterogêneo no Brasil, e seu impacto na mortalidade em nível populacional provavelmente varia de acordo com a infraestrutura regional de saúde e a disponibilidade de serviços especializados.^9,10^ As diretrizes clínicas atuais enfatizam que o diagnóstico precoce e o tratamento multidisciplinar são essenciais para reduzir a mortalidade.^9^ Ainda assim, a persistência de níveis elevados de mortalidade sugere que os benefícios desses avanços não estão distribuídos de forma uniforme no território nacional. O pico observado em 2004 pode refletir melhorias na detecção e notificação dos casos durante a fase inicial de consolidação do Sistema de Informações sobre Mortalidade (SIM), bem como flutuações temporais inerentes às análises baseadas em população.^23,24^

Notavelmente, a redução da mortalidade observada em 2020, seguida por um aumento parcial em 2021, deve ser interpretada no contexto da pandemia de COVID-19. Esse período foi marcado por interrupções no acesso aos serviços de saúde, atrasos no diagnóstico e possível classificação incorreta das causas de morte, especialmente para condições cardiovasculares. Estudos prévios no Brasil demonstraram que o excesso de mortalidade durante a pandemia pode estar associado à subnotificação, erros diagnósticos e à redução do acesso ao cuidado para condições não relacionadas à COVID-19.^25^

Nesse contexto, a análise espacial evidenciou diferenças importantes entre as regiões do país. Aglomerados de alta mortalidade foram observados principalmente nas regiões Centro-Oeste, Sul e Sudeste, com destaque para municípios como Corumbá de Goiás e São José do Rio Pardo. Esse cenário sugere a influência de fatores demográficos, como a maior proporção de população envelhecida nessas áreas, associada à maior prevalência de fatores de risco cardiovasculares. Adicionalmente, a maior capacidade diagnóstica e de notificação dos casos nessas regiões pode contribuir para as taxas mais elevadas observadas. O alto valor do I de Moran Global observado neste estudo pode refletir o uso de taxas suavizadas e o forte padrão de agrupamento regional no Brasil. Além disso, o uso de taxas médias populacionais pode ter contribuído para o aumento da estabilidade espacial e da autocorrelação. Também é importante considerar que valores extremos observados em municípios específicos podem ser influenciados por pequenos tamanhos populacionais, levando à instabilidade das taxas, bem como por estruturas locais de saúde ou padrões de notificação.^11^

Em contrapartida, nas regiões Norte e Nordeste, onde foram identificados aglomerados de baixa mortalidade, é plausível que os achados não reflitam necessariamente menor ocorrência da doença, mas sim subdiagnóstico e/ou sub-registro. Nessas regiões, o acesso a exames diagnósticos como o ecocardiograma, o qual é essencial para a avaliação da função miocárdica, ainda é limitado, e os sistemas de notificação de óbitos apresentam fragilidades.^7,14^ Nesse sentido, um estudo realizado no estado do Ceará demonstrou que municípios de menor porte populacional e com menor infraestrutura assistencial tendem a apresentar piores desfechos por doenças cardiovasculares, o que fortalece essa interpretação.^14^ Esses achados reforçam a necessidade de cautela na interpretação das taxas e evidenciam desigualdades regionais relevantes.

Essas desigualdades regionais reforçam a influência dos determinantes sociais da saúde e das fragilidades do sistema de atenção às doenças crônicas sobre os desfechos dos pacientes.^26^ Nesse contexto, destaca-se o papel da Atenção Primária à Saúde (APS) na vigilância e no rastreamento precoce das cardiomiopatias.^27^ Logo, a ampliação do acesso a exames diagnósticos, como o ecocardiograma, e o investimento na capacitação de profissionais da rede pública são estratégias recomendadas pelas diretrizes vigentes e potencialmente relevantes para melhorar os desfechos em nível nacional.^9^

Embora o presente estudo não tenha avaliado diretamente indicadores socioeconômicos específicos, como desigualdade de renda (por exemplo, coeficiente de Gini), Índice de Desenvolvimento Humano (IDH), gastos em saúde ou cobertura da atenção primária, esses fatores podem explicar parcialmente as disparidades regionais observadas. Estudos prévios no Brasil demonstraram que áreas com menor desenvolvimento socioeconômico e acesso reduzido aos serviços de saúde tendem a apresentar piores desfechos cardiovasculares, incluindo maiores taxas de mortalidade.^1-3^ Em particular, diferenças na cobertura da atenção primária e no acesso a recursos diagnósticos especializados podem contribuir para atrasos no diagnóstico e no tratamento, influenciando os padrões de mortalidade entre as regiões.^28-30^

Outra consideração importante é a heterogeneidade clínica e etiológica abrangida pelo código I42 da CID-10, que inclui diferentes subtipos de cardiomiopatia, como dilatada, hipertrófica, restritiva e formas secundárias, com mecanismos fisiopatológicos e prognósticos distintos.^3^ Quando analisada de forma agregada, essa heterogeneidade pode obscurecer padrões epidemiológicos específicos de cada subtipo e levar a interpretações simplificadas das tendências de mortalidade.

Ademais, devem ser considerados o subdiagnóstico e a subnotificação, especialmente em regiões com acesso limitado a ferramentas diagnósticas especializadas, como ecocardiografia e ressonância magnética cardíaca, bem como possíveis limitações na qualidade do preenchimento das declarações de óbito. As disparidades regionais no acesso a serviços de cardiologia e a variabilidade nos sistemas de informação em saúde podem contribuir para classificações incorretas e para uma maior proporção de causas de morte mal definidas.^31^

Adicionalmente, as mortes relacionadas às cardiomiopatias podem estar subestimadas devido ao registro frequente da insuficiência cardíaca (código I50 da CID-10) como causa básica de óbito, mesmo quando decorrente de cardiomiopatias (código I42 da CID-10). Essa limitação é reforçada pela incapacidade da plataforma DATASUS de identificar ou cruzar causas associadas de óbito.^31^

A cardiomiopatia chagásica, uma causa relevante de doença miocárdica no Brasil, é classificada sob um código diferente da CID-10 (B57.2) e pode não ser totalmente capturada em análises restritas ao código I42.^32^ Consequentemente, áreas identificadas com menores taxas de mortalidade podem refletir limitações na capacidade diagnóstica e na qualidade dos dados, em vez de uma real redução da carga da doença, exigindo cautela na interpretação de análises espaço-temporais.^31^

O uso de dados secundários representa uma limitação deste estudo e pode introduzir vieses relacionados à subnotificação, informações incompletas e inconsistências no registro das causas de morte.^32-34^ Análises baseadas em estimativas populacionais podem ser afetadas pela defasagem temporal do censo demográfico mais recente (2010), potencialmente influenciando a acurácia das estimativas da taxa de mortalidade.^34^ Como se trata de um estudo ecológico, os achados estão sujeitos ao viés de agregação e podem não refletir associações em nível individual.^35^ Em acréscimo, a análise considerou apenas a causa básica de óbito, o que pode subestimar a mortalidade relacionada às cardiomiopatias quando essas condições são registradas como causas associadas.^36^ A ausência de padronização por idade também pode limitar comparações entre regiões, considerando a forte dependência da mortalidade por cardiomiopatias em relação à idade e as diferenças regionais na estrutura etária da população.^37^ Por fim, o uso de taxas médias de mortalidade ao longo de um período prolongado pode obscurecer variações temporais e mudanças demográficas, potencialmente influenciando a interpretação dos padrões espaciais.^34^

Uma limitação adicional refere-se ao potencial de erro do tipo I devido a múltiplas comparações em análises espaciais envolvendo um grande número de municípios. A significância estatística foi estabelecida em p < 0,05 sem ajuste formal para múltiplos testes, como a correção pela taxa de descoberta falsa (*False Discovery Rate* – FDR). Embora essa abordagem possa aumentar a probabilidade de identificação de clusters espúrios, ela é comumente adotada em análises espaciais exploratórias.^38^ Logo, os padrões espaciais identificados devem ser interpretados com cautela.

Em síntese, a análise têmporo-espacial identificou áreas do território brasileiro com elevadas taxas de mortalidade por cardiomiopatias, evidenciando importantes desigualdades regionais. Dessa forma, os achados do presente estudo sugerem a necessidade de estratégias alinhadas à realidade e particularidades locais, voltadas para o fortalecimento da vigilância e do cuidado às cardiomiopatias. Nesse sentido, os resultados fornecem subsídios científicos para o planejamento e a organização de ações em saúde, com ênfase na Atenção Primária à Saúde (APS) principalmente nos locais com maior vulnerabilidade, visando ampliar o acesso e cuidado e, consequentemente, promover saúde e contribuir para a redução da morbimortalidade por cardiomiopatias no Brasil.

## Conclusão

Entre 2001 e 2021, a mortalidade por cardiomiopatias no Brasil apresentou redução temporal significativa, com diminuição média anual de 1,86%, embora tenha permanecido expressiva no período analisado. Observou-se maior frequência de óbitos entre homens, indivíduos com idade igual ou superior a 80 anos, pessoas autodeclaradas brancas, casadas e com menor escolaridade. A análise espacial evidenciou autocorrelação espacial positiva e identificou aglomerados de alta mortalidade predominantemente nas regiões Centro-Oeste, Sul e Sudeste, enquanto aglomerados de baixa mortalidade foram observados principalmente nas regiões Norte e Nordeste. Esses achados confirmam a heterogeneidade regional da mortalidade por cardiomiopatias no país e demonstram a utilidade da análise têmporo-espacial para a identificação de áreas prioritárias em saúde pública.
