# Supplementary material for: Análise Têmporo-Espacial da Mortalidade por Cardiomiopatias no Brasil entre 2001 e 2021
Source: Arq Bras Cardiol. 2026 Jul 15;123(6):e20260064. [Article in Portuguese] doi: 10.36660/abc.20260064 (PMC13399218; doi:10.36660/abc.20260064)
Supplement: Translation

# Translation: Spatiotemporal Analysis of Mortality from Cardiomyopathies in Brazil between 2001 and 2021

**DOI:** 10.36660/abc.20260064i

**Authors:** Luiz Fernando Kubrusly, Ana Clara Abdo Lopes, Fernanda Ritt de Souza, Gustavo Ferrucio Dieter, Laura Nadolny, Leonardo Moreira Dias, Letícia Moreira Dias, Luis Gustavo Machado Farias, Roberto Oliveira Basso, Vitória Naomi Okimura

**Affiliations:** 1 Brazil Faculdade Evangélica Mackenzie do Paraná, Curitiba, PR – Brazil; 2 Brazil Faculdade Pequeno Príncipe, Curitiba, PR – Brazil

**Keywords:** Cardiomyopathies, Mortality, Spatio-Temporal Analysis, Epidemiology

## Abstract

**Background:**

Brazil presents regional variations in mortality from cardiomyopathies, influenced by demographic characteristics, socioeconomic inequalities, and differences in access to health services. Between 2001 and 2021, 272,448 deaths from the disease were recorded in the country.

**Objective:**

To analyze, at the national level, the epidemiological profile and the spatiotemporal distribution of mortality from cardiomyopathies in Brazil, from 2001 to 2021.

**Methods:**

Epidemiological study based on secondary data from the Mortality Information System (SIM/DATASUS) and population estimates from IBGE. Crude mortality rates were calculated per municipality. Temporal analysis was performed using Joinpoint, estimating the annual percentage change (APC). Spatial analysis used GeoDa, with Bayesian smoothing and evaluation of spatial autocorrelation using the Global Moran’s I and LISA indices. Statistical significance was set at p < 0.05.

**Results:**

There were 272,448 deaths due to cardiomyopathies, predominantly in men (58.65%), individuals aged 80 or older (25.74%), white individuals (52.18%), married individuals (36.77%), and those with 1 to 3 years of completed schooling (21.91%). The average mortality rate was 6.66 per 100,000 inhabitants, reaching 7.64 per 100,000 in 2004 and falling to 4.15 per 100,000 in 2020. A significant annual reduction of 1.86% was observed (p < 0.05). Spatial analysis revealed clusters of high mortality, mainly in the Midwest, South, and Southeast regions, with Corumbá de Goiás standing out (49.34/100,000).

**Conclusion:**

There was a significant reduction in mortality from cardiomyopathies, despite the persistence of critical areas that require targeted strategies for surveillance, early diagnosis, and appropriate management.

## Introduction

Cardiomyopathies comprise a heterogeneous group of myocardial diseases characterized by structural and functional abnormalities that may progress to heart failure, malignant arrhythmias, and sudden cardiac death. According to the International Classification of Diseases (ICD-10), these conditions are classified under code I42. Over recent decades, a global increase in the prevalence of cardiomyopathies has been observed, particularly dilated cardiomyopathy, whose incidence ranges from 5 to 10 cases per 100,000 inhabitants per year. This trend reflects population aging and the growing burden of chronic non-communicable diseases.^1-3^

The etiology of cardiomyopathies is multifactorial and includes inflammatory, toxic, metabolic, and genetic causes, with particular emphasis on hereditary mutations, especially in hypertrophic cardiomyopathy.^3,4^ Traditional cardiovascular risk factors such as arterial hypertension, diabetes mellitus, obesity, and excessive alcohol consumption are also associated with the development and progression of these conditions.^5^

Clinically, patients may present with dyspnea, fatigue, palpitations, and peripheral edema, with manifestations ranging from asymptomatic forms in early stages to severe heart failure or sudden cardiac death.^6,7^ Diagnosis is based on clinical assessment combined with complementary tests, with echocardiography considered the gold standard for evaluating ventricular function and cardiac morphology.^8^ Treatment is directed toward the underlying etiology and includes both pharmacological and non-pharmacological therapies. Despite therapeutic advances, complications and mortality remain significant.^9,10^

In Brazil, studies have demonstrated marked regional variations in mortality from cardiomyopathies, suggesting the influence of demographic and socioeconomic factors, as well as inequalities in access to health services.^11-13^ However, most available investigations are limited by short time frames or isolated regional analyses, hindering an integrated understanding of the spatial distribution and temporal trends of cardiomyopathy-related mortality at the national level. ^14,15^

While previous studies often report regional differences using aggregated data, spatiotemporal analysis provides a more comprehensive approach by jointly evaluating variations across space and time. This allows the identification of persistent or emerging clusters and a better understanding of dynamic geographic patterns.^16,17^Additionally, methodological issues such as spatial aggregation (modifiable areal unit problem) and ecological bias may influence results, reinforcing the importance of appropriate analytical strategies in spatial epidemiology.^18^ Bayesian smoothing techniques and other rate-stabilizing approaches further improve estimate reliability in small areas by reducing random fluctuations.^19^

In this context, the present study aimed to analyze the epidemiological profile and the spatiotemporal distribution of mortality from cardiomyopathies in Brazil between 2001 and 2021. Secondary objectives included identifying spatial patterns and mortality clusters, as well as describing the distribution of deaths according to sociodemographic characteristics. Additionally, the place of occurrence of deaths was analyzed as a descriptive variable to complement the epidemiological profile.

## Methods

This was an epidemiological study based on the analysis of secondary health data, with a focus on spatiotemporal analysis. Data on deaths due to cardiomyopathies were obtained from the Mortality Information System (SIM) of the Department of Informatics of the Brazilian Unified Health System (DATASUS), Ministry of Health, accessed through the Tabnet platform, considering both place of residence and place of occurrence of death. Population data used as denominators for mortality rate calculations were obtained from DATASUS, based on IBGE population estimates by municipality, sex, and age for the period 2000–2021. These estimates are derived from demographic projection methods and intercensal adjustments performed by IBGE, using the 2010 national census as a reference to ensure consistency across the time series.

To analyze mortality trends in Brazil, the period from 2001 to 2021 was selected to allow a comprehensive assessment of the outcome over time. This was a census-based analysis, with no individual selection of participants, including all deaths from cardiomyopathies recorded in the SIM between 2001 and 2021. The population was defined as individuals of all ages. The variables analyzed included sex, age group, race/skin color, marital status, educational level, deaths by place of residence, year of death, place of death, and place of occurrence. Educational level categories followed the classification available in the DATASUS database and were maintained as originally provided to ensure consistency with national health information systems. The race/skin color variable was categorized according to the standardized classification adopted by DATASUS and the Brazilian Institute of Geography and Statistics (IBGE).

The underlying cause of death was defined according to the ICD-10 code I42. Epidemiological profile data and death frequencies were compiled using Microsoft Excel for Microsoft 365 MSO. Categorical variables were analyzed using absolute and relative frequencies. Annual mortality rates were calculated using the total number of deaths in each state as the numerator and the corresponding state population for each year as the denominator, expressed per 100,000 inhabitants. Municipality-level mortality rates were calculated using a population-averaging approach, defined as the mean number of deaths over the study period divided by the mean population between 2001 and 2021, expressed per 100,000 inhabitants. The mean population was obtained by summing annual population estimates over the study period and dividing by the number of years. This approach was adopted to reduce annual fluctuations and provide more stable estimates for small-area analyses; however, it does not account for differences in age structure across municipalities, which may influence comparisons.

The primary outcome of the study was mortality from cardiomyopathies, defined according to ICD-10 code I42. Initially, temporal trend analysis of annual mortality rates was performed (Figure 1). The annual percent change (APC) in mortality trends was estimated with a 95% confidence interval (95% CI), and statistical significance was set at p<0.05. Temporal trend analysis was performed using the Joinpoint Regression Program (version 5.0.2, National Cancer Institute, USA), using weighted least squares regression, estimating the annual percent change (APC) and 95% confidence intervals. A log-linear model was fitted, and the number of joinpoints was determined using permutation tests according to the software’s default settings. The final model selected included zero joinpoints (i.e., a single temporal trend). Subsequently, spatial analysis of cardiomyopathy mortality distribution was performed for each region of Brazil (Figure 2).

**Figure 1:** – Temporal pattern of mortality from cardiomyopathies in Brazil between 2001 and 2021. *Indicates that the Annual Percent Change (APC) is a significantly different from zero at the alpha = 0,05 level. Final Selected Model: 1 Joinpoint. Source: The authors (2025).

**Figure 2:** – Crude mortality rate from cardiomyopathies in Brazil, 2001–2021. Source: The authors (2025).

Spatial smoothing was performed using the local empirical Bayesian method to reduce random fluctuations in municipal mortality rates. A spatial weights matrix was constructed based on first-order queen contiguity, in which municipalities sharing at least one common boundary or vertex were considered neighbors.

Municipal boundaries were defined according to the official territorial division provided by the Brazilian Institute of Geography and Statistics (IBGE), without any modifications. Mortality data from DATASUS were linked to municipalities using official IBGE codes.

Sensitivity analyses using alternative neighborhood definitions (e.g., rook contiguity) were not performed. However, the use of queen contiguity was chosen due to its broader neighborhood structure, which is widely used in epidemiological spatial studies and provides more stable estimates in areas with heterogeneous territorial configurations.

Spatial analyses and thematic map construction were performed using GeoDa software, version 1.22.0.4 (2023). The categorization of map legends followed different approaches according to the type of analysis performed. For crude mortality rates (Figures 1 and 2), values were classified using quantiles, allowing a balanced distribution of municipalities across categories, and improving visualization and comparability. For the smoothed rates obtained through the local empirical Bayesian method (Figures 3–5), classification reflected the distribution of adjusted values, aiming to highlight spatial patterns after reducing random fluctuations. These classification strategies are commonly used in exploratory spatial analyses; however, different categorization methods may lead to variations in the visual interpretation of spatial patterns.

**Figure 3:** – Mortality rate from cardiomyopathies smoothed using the local empirical Bayesian method in Brazil, 2001–2021. Source: The authors (2025).

**Figure 5:** – Statistical significance of spatial clusters. Source: The authors (2025).

Spatial clusters were identified using the Global and Local Moran’s I indices. Global Moran’s I was used to test the hypothesis of spatial dependence by measuring the correlation among first-order neighbors, with values ranging from −1 to +1; values close to zero indicate the absence of spatial autocorrelation, with statistical significance defined as p<0.05. When spatial dependence was confirmed, Local Indicators of Spatial Association (LISA) were applied to identify local spatial clusters, also considering p<0.05. The results of these analyses were presented using Moran and LISA maps (Figures 4 and 5). The Moran map graphically represents the degree of similarity between neighboring areas and is divided into four quadrants:

**Figure 4:** – Spatial clusters of mortality from cardiomyopathies in Brazil, 2001–2021. Source: The authors 2025).

1. High–high (right superior quadrant): municipalities with high mortality rates surrounded by areas with similarly high rates
2. Low–low (left inferior quadrant): municipalities with low mortality rates surrounded by areas with low rates
3. High–low (right inferior quadrant): municipalities with high rates surrounded by low-rate neighbors
4. Low–high (left superior quadrant): municipalities with low rates surrounded by high-rate neighbors

The data used in this study are publicly available, aggregated, and do not allow individual identification; therefore, there is no potential for physical or moral harm to individuals or communities. Accordingly, the study was exempt from approval by a Research Ethics Committee and is in accordance with Resolution No. 466/2012 of the Brazilian National Health Council. Additionally, as the study is based on secondary data, the findings may reflect limitations related to data quality, such as underreporting and incomplete information in death certificates.

## Results

### Epidemiological profile

Between 2001 and 2021, a total of 272,448 deaths due to cardiomyopathies were recorded in Brazil. As shown in the Central figure, the sociodemographic characterization revealed a higher occurrence of deaths among male individuals and those aged 80 years or older. There was also a predominance of deaths among individuals classified as White, married, and with low educational attainment (1-3 years of schooling). The detailed epidemiological profile of deaths is presented in Table 1.

**Table 1:** – Epidemiological profile of individuals who died from cardiomyopathies in Brazil between 2001 and 2021

Variable	n	%
**Sex**		
Male	159,782	58.65
Female	112,618	41.33
Missing	48	0.02
**Age group (years)**		
<1	2,611	0.96
1-4	1,327	0.49
5-9	572	0.21
10-14	834	0.31
15-19	1,664	0.61
20-29	5,635	2.07
30-39	12,571	4.61
40-49	24,747	9.08
50-59	39,897	14.64
60-69	51,505	18.90
70-79	59,978	22.01
≥80	70,132	25.75
Age missing	975	0.36
**Race/ethnicity**		
White	142,166	52.18
Black	25,990	9.54
Brown	86,935	31.91
Asian	1,341	0.49
Indigenous	380	0.14
Missing	15,636	5.74
**Marital status**		
Single	66,998	24.59
Married	100,189	36.77
Widowed	66,280	24.33
Legally separated	15,681	5.76
Other	3,344	1.23
Missing	19,956	7.32
**Education (years)**		
None	39,515	14.50
1-3	59,684	21.91
4-7	56,125	20.60
8-11	29,699	10.90
≥12	10,933	4.01
Missing	76,492	28.08
**Place of death**		
Hospital	168,444	61.83
Other health facility	16,890	6.20
Home	72,962	26.78
Public place	4,852	1.78
Others	8,948	3.28
Missing	352	0.13

*Source: Department of Informatics of the Unified Health System (DATASUS), Ministry of Health, Brazil.*

Regarding the place of occurrence, most deaths occurred in hospital settings, followed by deaths at home, while the remaining categories showed lower relative frequencies (Table 1).

### Temporal analysis of mortality

The mean mortality rate from cardiomyopathies during the study period was 6.66 deaths per 100,000 inhabitants, with the highest rate observed in 2004 and the lowest in 2020. Temporal trend analysis demonstrated a significant reduction in mortality over the study period, with an average annual decrease of 1.86% (APC = −1.86%; 95% CI [−2.73; −1.03], p < 0.001). The final model did not include joinpoints, indicating a constant declining trend over time, as shown in Central Figure and Figure 1.

### Spatial analysis of mortality

Spatial analysis of crude mortality rates revealed marked heterogeneity in the distribution of deaths from cardiomyopathies across Brazil, with higher rates predominantly concentrated in the Midwest, South, and Southeast regions (Figure 2). Municipalities with the highest mortality rates during the period included Corumbá de Goiás, São José do Rio Pardo, Itajobi, Santo Inácio, and São Manuel.

After applying local empirical Bayesian smoothing, a clearer spatial pattern emerged, with clustering of municipalities presenting elevated mortality rates mainly in the Midwest and Southeast regions of Brazil (Figure 3). Spatial autocorrelation analysis confirmed the presence of significant spatial dependence, as evidenced by a positive Global Moran’s I (I = 0.864; p = 0.01).

Local spatial autocorrelation analysis (LISA) identified statistically significant spatial clusters. High–high clusters were mainly observed in the Midwest, South, and Southeast regions, whereas low–low clusters were predominantly located in the North and Northeast regions of the country (Central Figure, Figures 4 and 5).

## Discussion

The findings of this study demonstrate that, between 2001 and 2021, a total of 272,448 deaths due to cardiomyopathies were recorded in Brazil, predominantly among men (58.65%), individuals aged 80 years or older (25.74%), and those self-identified as White (52.18%). This epidemiological profile is consistent with data previously reported in the literature, which indicate greater severity and lethality of cardiomyopathies in older populations and in males, associated with cardiovascular aging and a higher burden of comorbidities, such as systemic arterial hypertension and diabetes mellitus.^2,5,20^

The higher mortality observed among men may be partly explained by biological and hormonal differences, as well as greater exposure to risk factors and a higher propensity for structural myocardial alterations and cardiovascular events, such as sudden cardiac death, which is consistent with findings from international studies and clinical guidelines.^2,6,7^ Furthermore, concerning age group, population aging is one of the main risk factors for the development of this condition, particularly in the case of dilated cardiomyopathy, due to increased myocardial vulnerability and the higher prevalence of the aforementioned comorbidities. This reinforces the relevance of this disease in countries undergoing advanced demographic transition, such as Brazil.^2,5,7,20^

Lower educational attainment was associated with higher mortality from cardiomyopathies: approximately 21.91% of individuals who died from this condition had 1 to 3 years of schooling. This finding is in line with that reported by Santos et al.,^14^ who suggest the influence of social determinants of health on cardiovascular outcomes, including reduced access to specialized healthcare services, delayed diagnosis, and lower therapeutic adherence. Additionally, the predominance of deaths among married individuals (36.77%) may reflect the greater longevity observed in this group, a phenomenon previously described in Brazilian demographic analyses.^21,22^

Regarding the place of death, most deaths occurred in the hospital setting (61.83%), which may indicate not only increased access to hospital admissions but also late diagnosis of cardiomyopathies, given that these conditions often go unnoticed in their early stages.^6^ Conversely, the significant proportion of deaths occurring at home (26.78%) suggests weaknesses in outpatient follow-up and in the early management of the disease, particularly in regions with major geographic and social constraints, as previously highlighted by Ziaeian and Fonarow.^2^

The observed reduction in mortality from cardiomyopathies (average annual decrease of 1.86%; p < 0.05) may be partially related to therapeutic advances in recent decades, including the use of angiotensin-converting enzyme inhibitors, beta-blockers, implantable devices, and heart transplantation. However, access to these therapies remains heterogeneous across Brazil, and their impact on population-level mortality likely varies according to regional healthcare infrastructure and availability of specialized services.^9,10^ Current clinical guidelines emphasize that early diagnosis and multidisciplinary treatment are essential to reduce mortality.^9^ Nevertheless, the persistence of high mortality levels suggests that the benefits of these advances are not evenly distributed across the national territory. The peak observed in 2004 may reflect improvements in case detection and reporting during the early consolidation of the Mortality Information System (SIM), as well as temporal fluctuations inherent to population-based analyses.^23,24^

Notably, the reduction in mortality observed in 2020, followed by a partial increase in 2021, should be interpreted in the context of the COVID-19 pandemic. This period was marked by disruptions in healthcare access, delays in diagnosis, and potential misclassification of causes of death, particularly for cardiovascular conditions. Previous studies in Brazil have demonstrated that excess mortality during the pandemic may be associated with underreporting, misdiagnosis, and reduced access to care for non-COVID conditions.^25^

In this context, spatial analysis revealed significant differences among the regions of the country. High-mortality clusters were observed mainly in the Central-West, South, and Southeast regions, with emphasis on municipalities such as Corumbá de Goiás and São José do Rio Pardo. This scenario suggests the influence of demographic factors, such as a higher proportion of elderly populations in these areas, combined with a greater prevalence of cardiovascular risk factors. Additionally, greater diagnostic capacity and more robust case reporting in these regions may contribute to the higher observed rates. The high value of Global Moran’s I observed in this study may reflect the use of smoothed rates and the strong regional clustering pattern in Brazil. Additionally, the use of population-averaged rates may have contributed to increased spatial stability and autocorrelation. It is also important to consider that extreme values observed in specific municipalities may be influenced by small population sizes, leading to rate instability, as well as by local healthcare structures or reporting patterns.^11^

In contrast, in the North and Northeast regions, where clusters of low mortality were identified, it is plausible that these findings do not necessarily reflect a lower occurrence of the disease, but rather underdiagnosis and/or underreporting. In these regions, access to diagnostic tests such as echocardiography (essential for the assessment of myocardial function) remains limited, and death notification systems present significant shortcomings.^7,14^ In this regard, a study conducted in the state of Ceará demonstrated that municipalities with smaller populations and less healthcare infrastructure tend to present worse outcomes for cardiovascular diseases, which supports this interpretation.^14^ These findings underscore the need for caution in interpreting mortality rates and highlight relevant regional inequalities.

Such regional disparities reinforce the influence of social determinants of health and weaknesses in the healthcare system for chronic disease management on patient outcomes.^26^ In this context, the role of Primary Health Care (PHC) in surveillance and early screening of cardiomyopathies is particularly noteworthy.^27^ Therefore, expanding access to diagnostic tests, such as echocardiography, and investing in the training of public healthcare professionals are strategies recommended by current guidelines and potentially relevant for improving outcomes at the national level.^9^

Although the present study did not directly evaluate specific socioeconomic indicators, such as income inequality (e.g., Gini coefficient), Human Development Index (HDI), healthcare expenditure, or primary healthcare coverage, these factors may partially explain the regional disparities observed. Previous studies in Brazil have demonstrated that areas with lower socioeconomic development and reduced access to healthcare services tend to present worse cardiovascular outcomes, including higher mortality rates.^1-3^ In particular, differences in primary health care coverage and access to specialized diagnostic resources may contribute to delays in diagnosis and treatment, influencing mortality patterns across regions.^28-30^

Another important consideration is the clinical and etiological heterogeneity encompassed by ICD-10 code I42, which includes distinct cardiomyopathy subtypes, such as dilated, hypertrophic, restrictive, and secondary forms, with different pathophysiological mechanisms and prognoses.^3^ When analyzed in an aggregated manner, this heterogeneity may obscure subtype-specific epidemiological patterns and lead to oversimplified interpretations of mortality trends.

Additionally, underdiagnosis and underreporting should be considered, particularly in regions with limited access to specialized diagnostic tools, such as echocardiography and cardiac magnetic resonance imaging, as well as potential limitations in the quality of death certificate completion. Regional disparities in access to cardiological services and variability in health information systems may contribute to misclassification and a higher proportion of ill-defined causes of death.^31^

Furthermore, cardiomyopathy-related deaths may be underestimated due to the frequent recording of heart failure (ICD-10 code I50) as the underlying cause of death, even when resulting from cardiomyopathies (ICD-10 code I42). This limitation is reinforced by the inability of the DATASUS platform to identify or cross-reference associated causes of death.^31^

Chagas cardiomyopathy, a relevant cause of myocardial disease in Brazil, is classified under a different ICD-10 code (B57.2) and may not be fully captured in analyses restricted to code I42.^32^ Consequently, areas identified with lower mortality rates may reflect limitations in diagnostic capacity and data quality rather than a truly reduced disease burden, requiring cautious interpretation in spatiotemporal analyses.^31^

The use of secondary data represents a limitation of this study and may introduce biases related to underreporting, incomplete information, and inconsistencies in the recording of causes of death.^32-34^ Analyses based on population estimates may be affected by the time lag of the most recent demographic census (2010), potentially influencing the accuracy of mortality rate estimates.^34^ As an ecological study, the findings are subject to aggregation bias and may not reflect individual-level associations.^35^ Furthermore, the analysis considered only the underlying cause of death, which may underestimate cardiomyopathy-related mortality when these conditions are recorded as associated causes.^36^ The absence of age standardization may also limit comparisons between regions, given the strong age dependence of cardiomyopathy mortality and regional differences in population age structure.^37^ Finally, the use of averaged mortality rates over a long period may obscure temporal variations and demographic changes, potentially influencing the interpretation of spatial patterns.^34^

An additional limitation relates to the potential for type I error due to multiple comparisons in spatial analyses involving a large number of municipalities. Statistical significance was set at p<0.05 without formal adjustment for multiple testing, such as False Discovery Rate (FDR) correction. Although this approach may increase the likelihood of identifying spurious clusters, it is commonly adopted in exploratory spatial analyses.^38^ Therefore, the identified spatial patterns should be interpreted with caution.

In summary, the spatiotemporal analysis identified areas of the Brazilian territory with high mortality rates from cardiomyopathies, highlighting significant regional inequalities. Accordingly, the findings of the present study suggest the need for strategies aligned with local realities and specificities, aimed at strengthening surveillance and care for cardiomyopathies. In this context, the results provide scientific evidence to support the planning and organization of health actions, with an emphasis on Primary Health Care (PHC), particularly in areas of greater vulnerability, intending to expand access to care and, consequently, promote health and contribute to the reduction of morbidity and mortality from cardiomyopathies in Brazil.

## Conclusion

Between 2001 and 2021, mortality from cardiomyopathies in Brazil showed a significant temporal decline, with an average annual reduction of 1.86%, although it remained substantial throughout the study period. A higher frequency of deaths was observed among men, individuals aged 80 years or older, those who self-identified as White, married individuals, and those with lower levels of education. Spatial analysis revealed positive spatial autocorrelation and identified clusters of high mortality predominantly in the Central-West, South, and Southeast regions, while clusters of low mortality were mainly observed in the North and Northeast regions. These findings confirm the regional heterogeneity of cardiomyopathy-related mortality in the country and demonstrate the usefulness of spatiotemporal analysis for identifying priority areas in public health.
